# Supplementary material for: Liquid Biopsy for Disease Monitoring in Non-Small Cell Lung Cancer: The Link between Biology and the Clinic
Source: Cells. 2021 Jul 28;10(8):1912. doi: 10.3390/cells10081912 (PMC8394732; doi:10.3390/cells10081912)
Supplement: Supplementary file 1 [file cells-10-01912-s001.zip › cells-1312213-supplementary.pdf]

SUPPLEMENTARY DATA

1. Material and Methods

1.1. Blood Sampling, Cell-Free DNA Extraction and Quantification

Sampling was obtained before patients had received any cancer treatment. Blood samples were collected in K2EDTA BD Vacutainer® PPT™ Plasma Preparation Tube (Becton Dickinson, Franklin Lakes, NJ, USA) and shipped at room temperature to the molecular laboratory in a time-lapse less than 4 h and plasma was immediately isolated upon receipt by centrifugation. Blood samples were centrifuged for 10 min at 1200 rpm, and the plasma supernatant was centrifuged for 10 min at 3000 rpm, then stored at −80C. Cell free DNA was extracted from aliquots (1 mL) of plasma with the MagMax Cell-Free Total Nucleic Acid Isolation Kit (Thermo Fisher Scientific, Waltham, MA, USA) and quantified with the dsDNA HS assay kit using a Qubit 3.0 or 4.0 Fluorometer (Thermo Fisher Scientific), following the manufacturer's instructions.

1.2. Library Preparation

Targeted-plasma NGS was performed using a validated amplicon-based NGS Oncomine™ Lung cfDNA Assay (Thermo Fisher Scientific, Waltham, MA) that uses target gene enrichment using a PCR with a set of primers for exons of selected genes, covering more than 150 hotspots on the following eleven genes: *ALK, BRAF, EGFR, ERBB2, KRAS, MAP2K1, MET, NRAS, PIK3CA, ROS1,* and *TP53*.

1.3. Next-Generation Sequencing, Variant Calling, and Bioinformatic Analysis

The libraries were sequenced with either the Ion PGM or Ion S5XL sequencer using semiconductor sequencing technology, following the manufacturer's instructions. The sequencing reads were aligned to the human reference hg19 genome with the Ion Torrent Suite V3.4.2 (or Torrent Suite Software™ v5.8). Accepted metrics for each sample were number of reads per sample, > 2,500,000 reads (for Oncomine™ Lung cfDNA Assay libraries); on-target reads, > 90%; read uniformity, > 90%; mean depth, > 20,000x. Coverage metrics for each amplicon were obtained by running the Coverage Analysis Plugin software v5.6.1 and later (Thermo Fisher Scientific). Polymorphisms and synonymous or intronic mutations were excluded. The Catalogue of Somatic Mutations in Cancer (COSMIC) database was used to access the clinically relevant variants.

1.4. Plasma NGS sequencing analysis

Table S1. Patient characteristics and NGS plasmatic and tissue results at baseline.

Case ID #	Gender	Age	Smoking Status	Morphology	Tumour stage (TNM)	Genomic Profile		Tumour	ctDNA
						Gene	A.a	AF%	
**2**	F	38	S	Adenocarcinoma	T2aN2M1b	EGFR	c.2240_2257 del18	40.0	0.72
**15**	M	60	ExS	Adenocarcinoma	T4N2M1c	KRAS	c.182A > G	8.8	0.43
						TP53	c.344G > T	25.0	0.71
						STK11	c.597G > T	12.8	0.53
**62**	M	60	NS	Adenocarcinoma	T1aN0M1a	BRAF	c.1799T > A	36.3	0.53
**74**	M	59	ExS	Adenocarcinoma	T4N2M1c	EGFR	c.2236_2250 del15	11.1	0.27
						KRAS	c.182A > G	0.38	0.07
**80**	F	58	NS	Adenocarcinoma	T4N3M1c	BRAF	c.1799T > A	50.4	9.9
						TP53	c.476C > G	39.2	4.9

**81**	M	40	NS	Adenocar-cinoma	TxN0M1a	EGFR	c.2236_2250 del15	36.7	0.18
						EGFR	c.2369C > T	15.0	0.14
**89**	M	64	NS	Adenocar-cinoma	T4N3M1a	EGFR	c.2237_2255 del18InsC	nq	0
**91**	F	76	NS	Adenocar-cinoma	T4N2M1a	EGFR	c.2573T > G	nq	1,72
							C.2369C > T	0	0.28
**95**	F	59	NS	Adenocar-cinoma	T2bN1M1c	EGFR	c.2240_2257 del18	96.50	0.65
							c.2369C > T	0	0
**107**	F	54	NS	Adenocar-cinoma	T3N2M1c	EGFR	c.2240_2257 del18	67,20	5.50
							c.2369C > T	0.60	1.25
**130**	M	63	NS	Adenocar-cinoma	T1bN1M1b	EGFR	c.2235_2249 del15	13.20	1.06

NS—Non-smoker, S—smoker, Ex—smoker, nq—not quantified

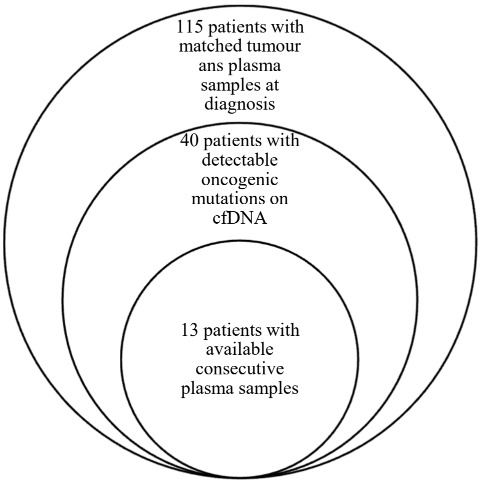

Figure S1. Study Diagram. Among a cohort of 115 patients with advanced lung adenocarcinoma, 40 patients had detectable oncogenic alterations. The most frequent alterations were *n* = 20 EGFR, *n* = 17 KRAS, *n* = 5 TP53, *n* = 4 BRAFV600E. Patients with consecutive plasma samples (*n* = 10 EGFR, *n* = 2 BRAF V600E, and *n* = 1 KRAS/TP53/STK11) were included in this study.

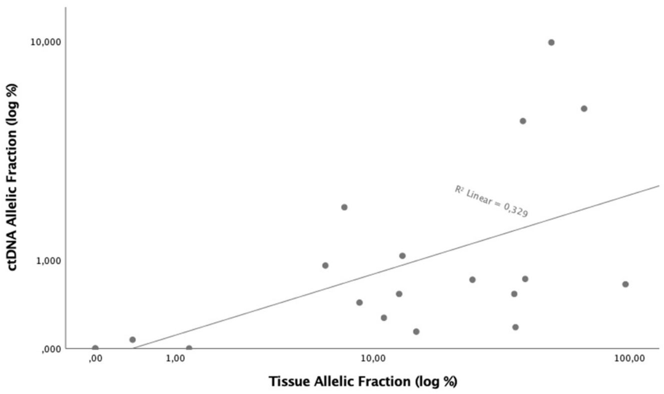

Figure S2. Comparison between VAF detected in tissue DNA and ctDNA.
